# Value of Contrast-Enhanced Ultrasound in the Ultrasound Classification of Cervical Tuberculous Lymphadenitis

**DOI:** 10.3389/fmed.2022.898688

**Published:** 2022-06-14

**Authors:** Ying Zhang, Tianzhuo Yu, Dongming Su, Wei Tang, Gaoyi Yang

**Affiliations:** Department of Ultrasonography, Affiliated Hangzhou Chest Hospital, Zhejiang University School of Medicine. Zhejiang Integrated Traditional and Western Medicine Hospital, Hangzhou Red Cross Hospital, Hangzhou, China

**Keywords:** contrast-enhanced ultrasound, tuberculous lymphadenitis, classification, cervical, diagnosis

## Abstract

**Purpose:**

The purpose of this study was to investigate the clinical value of contrast-enhanced ultrasound (CEUS) in the ultrasound (US) classification of cervical tuberculous lymphadenitis (CTL).

**Materials and Methods:**

This retrospective study included 70 patients diagnosed with CTL. All patients underwent both conventional US and CEUS. Both methods were compared to determine their agreement with pathological CTL results.

**Results:**

The results of conventional US classification were as follows: 18 patients (25.7%) were type I, 25 patients (35.7%) type II, 21 patients (30.0%) type III, and 6 patients (8.6%) type IV, respectively. The results of CEUS classification were as follows: 9 patients (12.9%) were type I, 33 patients (47.1%) type II, 22 patients (31.4%) type III, and 6 patients (8.6%) type IV. Conventional US classification and pathological results showed moderate agreement in terms of US classification results for CTL (*Kappa* = 0.693); the accuracy of conventional US classification was 78.6% (55/70), and the accuracy of types II and III were 71.0% (22/31) and 82.6% (19/23), respectively. CEUS classification and pathological results showed strong agreement (*Kappa* = 0.871); the accuracy of CEUS classification was 91.4% (64/70), and the accuracy of types II and III were 93.6% (29/31) and 87.0% (20/23), respectively.

**Conclusion:**

In combined with conventional US, CEUS could provide more information on blood flow enhancement patterns and identify the area of lymph node necrosis in CTL. This could contribute to a more accurate US classification of CTL.

## Introduction

Tuberculosis is an infectious disease caused by *Mycobacterium tuberculosis* (MTB) complex (1). Approximately a quarter of the world’s population is infected with MTB (2). Extrapulmonary tuberculosis (EPTB) accounts for approximately 8–24% of all tuberculosis cases and comprises a considerable portion of the tuberculosis epidemic (2). Early detection and timely treatment of EPTB are crucial aspects of the World Health Organization’s strategy against tuberculosis (3). Tuberculous lymphadenitis is the most presentation form of EPTB and often occurs in the cervical region with atypical clinical manifestations. These are frequently missed during diagnosis or misdiagnosed, thereby delaying the timely treatment (3–5). At present, cervical tuberculous lymphadenitis (CTL) treatment involves oral administration of anti-tuberculosis chemotherapy. In case of abscess or sinus tract formation, interventional therapy or surgical resection is also be required (6). Therefore, early diagnosis and classification of CTL are of great significance to the clinical treatment plan. CTL classification through computed tomography (CT) has been investigated by Lee et al. since as early as 1994 (7), however, studies on the use of contrast-enhanced ultrasound (CEUS) for CTL classification are limited.

Previous studies have reported that CEUS is an effective method to examine microvascular perfusion in lesions, and it has significantly improved our ability to identify necrotic areas. The blood flow enhancement patterns, diagnosis, and differential diagnosis of CTL have been intensively studied (8–10). Furthermore, CEUS has been used to diagnose and classify pancreatic cystic lesions, cystic renal tumors, and other diseases (11, 12). In the present study, in addition to conventional US for the classification of CTL, CEUS was used to re-examine the lesion. In addition, the CEUS results were compared with the pathological results to explore the importance of CEUS in the US classification of CTL.

## Materials and Methods

### Patients

This retrospective study was conducted at Hangzhou Red Cross Hospital (Zhejiang Tuberculosis Diagnosis and Treatment Center) and included the data of 70 patients with CTL who were treated at the hospital from June 2020 to December 2021. All cases of CTL were caused by MTB complex.

The inclusion criteria were as follows: (1) underwent lymph node ultrasound-guided core needle biopsy or excision biopsy to obtain specimens; (2) positive acid-fast bacilli (AFB) smear or MTB culture; (3) complete imaging data, included conventional US and CEUS examinations; (4) no history of anti-tuberculosis chemotherapy. Patients were excluded if they did not undergo invasive procedures or did not take the relevant tests and could not be classified based on pathological results. The use of the patient database was approved by the Hospital Ethics Committee. The need for patient consent was waived due to the retrospective nature of the study. The images for each patient were obtained in JPG and AVI format from the PACS database, and patient information was collected from the hospital records.

Conventional US and CEUS images were analyzed by two radiologists with 10 years of diagnostic experience who were blinded to the pathological results. Conflicting findings were resolved through discussion among the experts till a consensus was reached.

### Ultrasound Examination

We used an iU22 ultrasound diagnostic instrument (Philips Healthcare, Bothell) with L12–5, and L9–3 probes, and the corresponding frequency was 5–12 MHz, and 3–9 MHz, respectively.

Patients were placed in the supine or lateral position, and each area was checked according to the process for corresponding anatomical regions. The largest suspicious lymph node was selected as the object under observation. The lymph node size, border, capsule, lymph hilum, internal echo, cystic necrosis, calcification, blood flow signals, and lymph node surroundings were observed. According to the pathological findings and conventional US findings, CTL were classified into four types (13, 14) as follows: (1) nodular type (type I): an enlarged lymph node with a clear border, intact capsule, thickened cortex, uniform internal echoes, visible hilum of lymph node, hilar blood flow, and pathological findings were mainly lymphocytes and tuberculous granulomas; (2) inflammatory type (type II): an enlarged lymph node with a clear border, thickened cortex, uneven internal echoes, some echoless areas, narrowed or absent hilum of lymph node, partial fusion of the lymph node, signs of inflammation in the surrounding area, reduced blood flow or no sign of blood flow in the lymph node, and pathological findings were mainly caseous necrosis or inflammatory cell infiltration; (3) abscess and sinus tract type (type III): a significantly enlarged lymph node with a blurred border, irregular shape, chaotic internal echoes, absent hilum of lymph node, partially destroyed capsule, a sinus tract caused by skin impairment, peripheral or mixed blood flow signals, and pathological findings were mainly liquefactive necrosis; and (4) healed and calcified type (type IV): a shrunken lymph node with a clear border, low internal echoes, strong calcification echoes or strong fibrotic cord-like echoes, no blood flow signals, and pathological findings were mainly fibrous hyperplasia.

### Contrast-Enhanced Ultrasound Examination

CEUS examination low mechanical index (0.06) pulse reverse harmonic imaging and the sulfur hexafluoride microbubble ultrasonic contrast agent SonoVue (Milan, Italy, Bracco SpA) were used for patient examination. Following intravenous injection of 2.4 mL contrast agent, dynamic observation of lymph node enhancement and continuous observation was performed for 2 min. The images were stored in the instrument hard-disk for subsequent analysis. Using CEUS patterns were classified as homogeneous enhancement, septum-like enhancement, annular enhancement and no enhancement. Homogeneous enhancement was defined as simultaneous arrival of contrast agents in different parts of the same lymph node. Septum-like enhancement referred to the internal rendering into a partition or honeycomb enhancement, and annular enhancement was defined as the edge and surrounding (9).

### Statistical Analysis

The data were analyzed by SPSS 23.0 statistical software (United States, IBM). Measurement data were expressed as mean ± SD; the count data were expressed as the number and percentage of cases. Conventional US and CEUS were used for US classification and the agreement of both methods with the pathological results of CTL were compared. Kappa result be interpreted as follows: values 0–0.20 as none and 0.21–0.39 as minimal, 0.40–0.59 as weak, 0.60–0.79 as moderate, 0.80–0.89 as strong, and 0.90–1.00 as almost perfect agreement.

## Results

### Patients

Of the 70 patients with CTL, 34 were males and 36 were females (aged 18–76 years, 39.21 ± 16.85 years). The mean long-axis diameter of the lymph node was 2.96 ± 1.18 cm (range, 1.1–5.9 cm) and the short-axis diameter was 1.48 ± 0.64 cm (range, 0.6–3.3 cm) for all patients.

### Conventional Ultrasound and Contrast-Enhanced Ultrasound Classification Results

**FIGURE 1:**
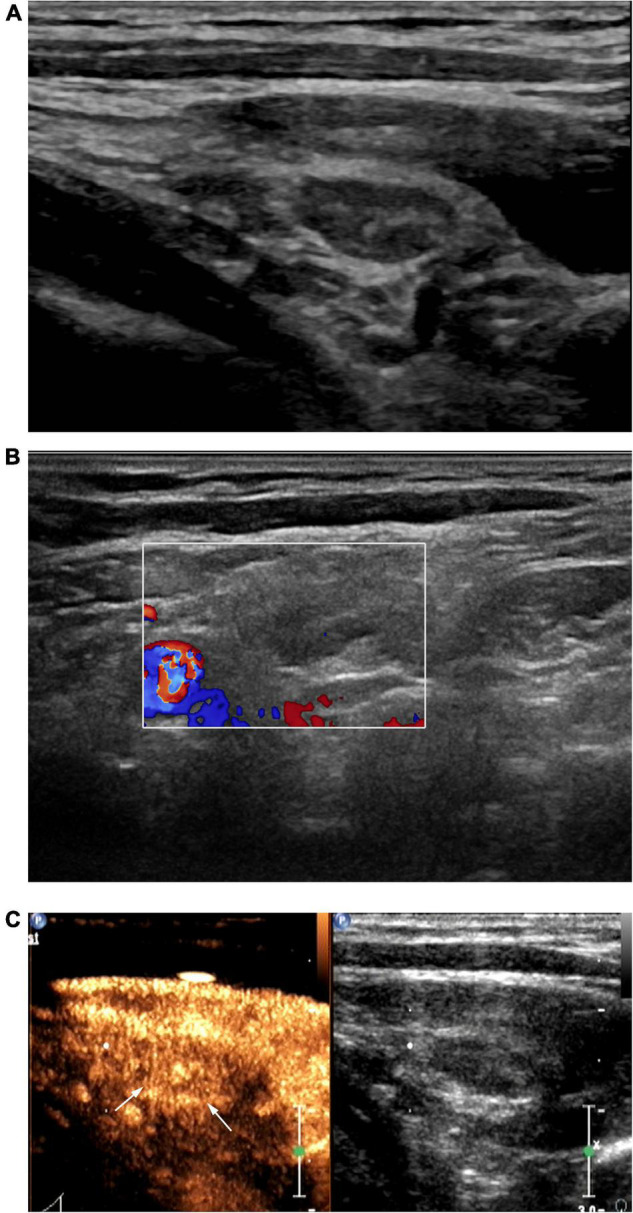
Patient with left cervical tuberculous lymphadenitis (CTL) (30-year-old man). Conventional ultrasound (US) showed type I cervical tuberculous lymphadenitis (CTL), which remains classified as type I after contrast-enhanced ultrasound (CEUS). US-guided fine-needle aspiration was performed to obtain a small amount bloody fluid. Pathological analysis of this was consistent with the manifestations of type I CTL. **(A)** Two-dimensional US indicated a lymph node 1.1 × 0.5 cm in size with a clear border, thickened cortex, uniform internal echoes, visible hilum of lymph node. **(B)** Punctate blood flow signals were observed in the center of the lymph node. **(C)** Under peak CEUS enhancement, homogeneous enhancement of the lymph node was observed.

**FIGURE 2:**
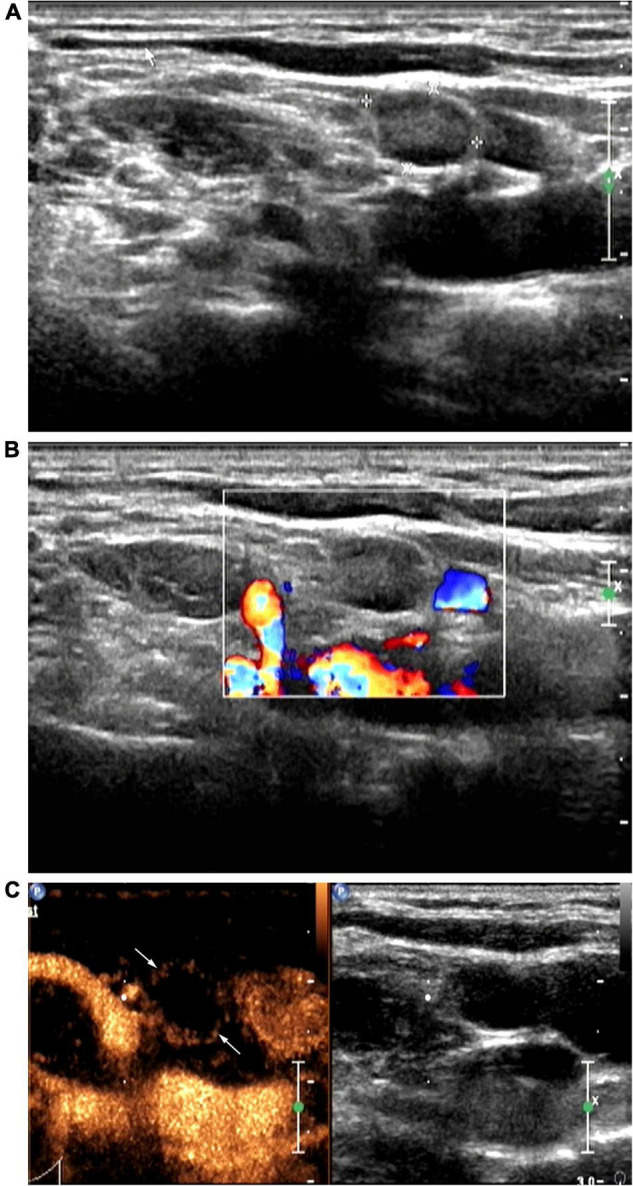
Patient with right cervical tuberculous lymphadenitis (CTL) (28-year-old man). Conventional ultrasound (US) showed type II CTL, which was re-classified as type III after contrast-enhanced ultrasound (CEUS). US-guided fine-needle aspiration was performed to obtain a small amount of pus. Pathological analysis of this was consistent with the manifestations of type III CTL. **(A)** Two-dimensional US indicating a lymph node (size, 1.0 × 0.7 cm) with a clear border, thickened cortex, uneven internal echoes, the hilum of lymph node was unclear. **(B)** No color blood flow signals. **(C)** Under peak CEUS enhancement, annular enhancement of the lymph node was observed, and a large area within the lymph node showed no enhancement.

Using conventional US, 18 (25.7%), 25 (35.7%), 21 (30.0%), and 6 (8.6%) patients were classified under types I (Figures 1A,B), II (Figures 2A,B), III and IV CTL, respectively. Using CEUS, 9 (12.9%), 33 (47.1%), 22 (31.4%), and 6 (8.6%) patients were categorized as types I, II, III and IV CTL, respectively.

CEUS revealed homogeneous enhancement and septum-like enhancement in 50.0% (9/18) (Figure 1C) and 50.0% (9/18), respectively, of the patients diagnosed with type I CTL by conventional US. Moreover, CEUS revealed that septum-like, annular, and no enhancement were more common among patients with CTL types II and III diagnosed using conventional US, with each accounting for 88.0% (22/25), 8.0% (2/25), 4.0% (1/25) among type II patients, respectively, and 9.5% (2/21), 57.2% (12/21) (Figure 2C), 33.3% (7/21) among type III patients, respectively.

**FIGURE 3:**
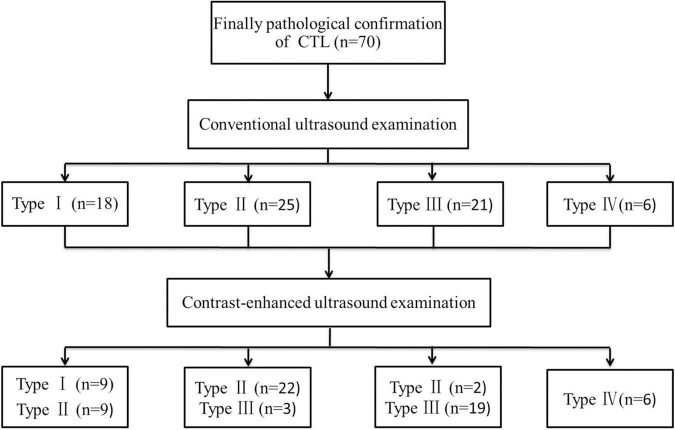
Cervical tuberculous lymphadenitis classification results of conventional ultrasound using contrast-enhanced ultrasound.

Using conventional US for the classification of CTL, CEUS was used to re-examine, which was re-classified after CEUS. These cases were re-classified is shown in the Figure 3.

### Concordance Analysis

Of the 70 patients with CTL, the pathological results were mainly lymphocytes and granulomatous lesions in 10 cases (corresponding to US type I), coagulative necrosis in 31 cases (corresponding to US type II), liquefactive necrosis and granulomatous lesions in 23 cases (corresponding to US type III), and fibrous hyperplasia in 6 cases (corresponding to US type IV).

The results of conventional US classification were as follows: 18 patients (25.7%) with type I, 25 (35.7%) with type II, 21 (30.0%) with type III, and 6 (8.6%) with type IV, respectively. Using CEUS classification, 9 patients (12.9%) were identified as type I, 33 (47.1%) as type II, 22 (31.4%) as type III, and 6 (8.6%) as type IV (Table 1).

**TABLE 1:** Comparison of conventional US and CEUS classification results with pathological results (number).

Classification	Pathological results			
	Type I (*n* = 10)	Type II (*n* = 31)	Type III (*n* = 23)	Type III (*n* = 6)
**Conventional US**				
Type I	9	6	3	0
Type II	1	22	1	1
Type III	0	2	19	0
Type IV	0	1	0	5
**CEUS**				
Type I	9	0	0	0
Type II	1	29	3	0
Type III	0	2	20	0
Type IV	0	0	0	6

*US, ultrasound; CEUS, contrast-enhanced ultrasound.*

Conventional US showed moderate agreement with the pathological results in terms of US classification of CTL (*Kappa* = 0.693), with a 78.6% (55/70) accuracy of conventional US classification; the accuracy of type II and type III classification were 71.0% (22/31) and 82.6% (19/23), respectively. Conversely, CEUS showed strong agreement with the pathological results (*Kappa* = 0.871); the accuracy of CEUS classification results was 91.4% (64/70). Moreover, the accuracy of type II and III classification were 93.6% (29/31) and 87.0% (20/23), respectively.

## Discussion

Tuberculous lymphadenitis is a common cause of lymphadenopathy in areas where tuberculosis is endemic, and accounts for 35% of all EPTB cases (4, 15). It is considered to be the local manifestation of the systemic disease that has disseminated to local lymph nodes (16). Most patients with CTL usually present with unilateral, multiple, matted neck non-tender swelling, without systemic symptoms and there are several infectious and non-infectious diseases that can mimic the same clinical picture (16). Therefore, patients with tuberculous lymphadenitis often have a poor prognosis due to delays in the diagnosis and treatment (5), early diagnosis and selection of an appropriate regimen of therapy are essential.

US is an important method that is used to examine cervical lymph nodes, which can accurately demonstrate the sites, pattern and extend of the disease, and US findings also show a good correlation with the pathological results of tuberculosis (10, 17, 18). In tuberculous lymphadenitis, MTB is engulfed by macrophages to elicit an immune response, which results in lymphadenosis and produces the typical tuberculous granulomas and caseous necrosis (19, 20). Different lesion types can manifest at different stages of the disease. In the early stage of the disease, conventional US shows no obvious changes in the internal structure and blood flow of lymph nodes (21). As the disease progress, tuberculous granulomas would form, followed by caseous necrosis. At this point, the lymph node cortex thickens and the hilum shrinks or disappears, and blood flow to the lymph node decreases (21, 22). In the later stages, lesions liquefy to form abscess or sinus tract. Chaotic echoes are noted within the lymph node, and the hilum of lymph node disappears (21, 22). With effective treatment, MTB is inhibited and the lesions improve and undergo calcification, leading to enhanced immunity (15). These stages of tuberculous lymphadenitis are consistent with the pathological processes observed on CT (22).

Three patterns of tuberculosis lymphadenitis have been reported on contrast-enhanced CT. First, in the early course of disease, the node is homogeneously enhanced. Second, as the disease progresses, the most common pattern is observed: a node with a central area of necrosis, which manifests as annular enhancement. The third pattern is a homogenous fibro-calcified node with noticeable calcification (15). This is consistent with the CEUS findings of this study. In our study, CEUS mostly showed homogeneous enhancement or septum-like enhancement on the early stage of CTL. Thereafter, some lymph nodes show no enhancement in the central area characterized by annular enhancement. Healed and calcified type is usually seen in treated patients. According to prior studies, CEUS has been supposed to be a promising and less invasive method which is based on the application of intravenous contrast agent. In some cases, the nature of CTL lesion could be predicted through the characterization of blood perfusion reflected by CEUS (9, 10, 23). In our study, the US classification of CTL was further improved by the blood perfusion pattern of CEUS.

At present, CTL is mainly treated using systematic application of anti-tuberculosis chemotherapy, the Infectious Disease Society of America (IDSA) guideline recommends surgical excision only in unusual circumstances, disease caused by drugresistant organism, or paradoxical upgrading reactions (6, 24, 25). CTL with different stages of procession require different treatment methods, type I and type IV are the early and healing phases for the lesion, respectively, and systematic anti-tuberculosis chemotherapy can be used, while there are diverse clinical interventions for types II and III CTL, such as tuberculous abscess that has not ruptured can be subjected to puncture and aspiration. In a large retrospective cohort study, 31% of patients were treated with needle aspiration of tuberculous nodes, and with most patients having a good clinical response (26). However, management of very fluctuant tuberculous nodes is unclear. A percentage of these nodes will become adherent to the overlying skin and, even on effective anti-tuberculosis chemotherapy, result in spontaneous formation of a sinus tract (26). In case of sinus tract formation, drainage and dressing change or radical surgical removal of the lesion may be performed as clinical intervention (6, 24, 25). In this study, according to the results of CTL US typing, it may be provided more information for the selection of treatment methods. Therefore, obtaining an accurate classification is vital for the selection of appropriate treatment method and for the determination of surgical requirements. However, the various pathological features of CTL correspond to diverse and complex imaging manifestations, this makes US classification challenging.

Recent research using superb microvascular imaging (SMI) to study US classifications of CTL has found SMI results to be in perfect agreement (*Kappa* = 0.948) with pathological results, with an accuracy of 96.2% (13). Furthermore, studies have reported good agreement between SMI and CEUS findings, suggesting the potential use of CEUS for US classification of CTL (27). In our study, CEUS showed annular enhancement and no enhancement in 12.0% of the 25 patients diagnosed with type II CTL by conventional US, resulting in the reclassification of these cases as type III CTL. CEUS showed a septum-like enhancement in 9.5% of the 21 patients diagnosed with type III CTL by conventional US. These cases were re-classified as type II CTL. The Kappa values for the agreement of conventional US and CEUS classification results with the pathological results were 0.693, 0.871, respectively, and the accuracy were 78.6%, 91.4%, respectively. The accuracy of type II CTL classified by conventional US was 71.0%, and that by CEUS was 93.6%. This demonstrated that classification by CEUS had better agreement with pathology results than conventional US. Therefore, CEUS is a valuable tool for US classification of CTL. This may be attributable to the thin blood vessels and low flow velocity in normal lymph nodes. When caseous necrosis or liquefactive necrosis occurs, MTB often accumulates in the hilum of the lymph node, destroying the normal vascular structure and resulting in an insufficient blood supply to the center of the lymph node (9). Conventional US cannot easily distinguish between caseous necrosis and poor lymphatic microcirculation (23, 28). However, CEUS is more sensitive to lymphatic lesions and areas of necrosis with no enhancement (29). Well-known, US is easily available and frequently used for imaging lymph nodes. CTL may show internal septations, solid components, or areas of necrosis, with vascularity of these elements assessable with color Doppler. However, vascularity in fine septations and areas of necrosis may be challenging to resolve for conventional US. Higher-contrast resolution of CEUS could help identify details with CTL (solid nodules, septa, and areas of necrosis). Graumann et al. (30) report in a prospective cohort study, comparison of CT, CEUS, and MR for categorizing complex renal cystic masses, because of better contrast resolution, CEUS has superior results to CT and similar results to MR imaging, when comparing delineation of solid and cystic components. CEUS gives an opportunity to image tissue vascularity in real-time, rapid, and continuous fashion over several minutes and US contrast agents have the benefit of being safe and with less severe adverse effects (12). In addition, compared with CT and MR, CEUS is less expensive, radiation-free, and more easily accepted by patients. Therefore, in the present study, the combined use of conventional US and CEUS was able to visualize both microvascular perfusion of lymph nodes and the necrotic areas of lesions, this could contribute to the improvement of US classification of CTL.

However, our study still has certain limitations. First, as this was a retrospective study, there may be some sampling biases. Second, as the positive rate of AFB smear and MTB culture in this study was low, the sample size of our study was limited. Further multicenter studies with larger samples are warranted to verify our results. Third, intra-observer consistency was not assessed, which may lead to the unclear retribution. Fourth, the assessment was limited because of the lack of other medical imaging data, such as CT or MRI. Fifth, Since we are a tuberculosis center in Zhejiang Province, where tuberculosis is very clustered, radiologist are more familiar with the CEUS images of CTL, as a result, the actual diagnostic consistency and accuracy might have been overestimated in our analysis. Finally, this study was conducted in a high tuberculosis burden area, and therefore it may not be applicable to other areas.

## Conclusion

In summary, combining conventional US with CEUS improves the evaluation of different stages of tuberculous lymphadenitis and the enhancement patterns provide richer information for the US classification of CTL. Therefore, CEUS is a valuable supplementary method for the US classification of CTL. Combining US classification of CTL with a comprehensive analysis of the clinical manifestations and laboratory test results provides a more accurate assessment of the stage of the disease and may be provided more information for the selection of treatment methods.

## Data Availability Statement

The data analyzed in this study is subject to the following licenses/restrictions: The datasets generated and/or analyzed during the current study are not publicly available due to an IRB decision which was made in the interest of ensuring patient confidentiality but are available from the corresponding author on reasonable request. Requests to access these datasets should be directed to YZ, zhangying5201@163.com.

## Ethics Statement

The studies involving human participants were reviewed and approved by the Hangzhou Red Cross Hospital Ethics Committee. Written informed consent for participation was not required for this study in accordance with the national legislation and the institutional requirements. Written informed consent was not obtained from the individual(s) for the publication of any potentially identifiable images or data included in this article.

## Author Contributions

YZ, GY, and TY involved in the study conception, the study design, data management, and planning the analysis. YZ, DS, and WT implemented the study. YZ wrote the first draft. All authors read and approved the final manuscript and involved in interpreting the data.

## Conflict of Interest

The authors declare that the research was conducted in the absence of any commercial or financial relationships that could be construed as a potential conflict of interest.

## Publisher’s Note

All claims expressed in this article are solely those of the authors and do not necessarily represent those of their affiliated organizations, or those of the publisher, the editors and the reviewers. Any product that may be evaluated in this article, or claim that may be made by its manufacturer, is not guaranteed or endorsed by the publisher.
